# Integrative Analysis Identified a 6-miRNA Prognostic Signature in Nasopharyngeal Carcinoma

**DOI:** 10.3389/fcell.2021.661105

**Published:** 2021-07-16

**Authors:** Yunqin Chen, Zhen Wang, Hong Li, Yixue Li

**Affiliations:** ^1^School of Life Sciences and Biotechnology, Shanghai Jiao Tong University, Shanghai, China; ^2^Bio-Med Big Data Center, CAS Key Laboratory of Computational Biology, Shanghai Institute of Nutrition and Health, University of Chinese Academy of Sciences, Chinese Academy of Sciences, Shanghai, China

**Keywords:** nasopharyngeal carcinoma, miRNA signature, prognosis biomarker, machine learning method, precision treatment

## Abstract

**Background:**

Nasopharyngeal carcinoma (NPC) is an Epstein–Barr virus-associated epithelial malignancy, which is rare in America but endemic in China. The current clinical gold TNM-based standard for prognosis may not be enough. Although some studies have reported that some miRNAs have a prognostic power in NPC, there is a scarcity of independent validation for these miRNAs.

**Methods:**

In this work, we firstly conducted a literature review of all miRNA profiling datasets with survival information, then integrated miRNA expression data across different profiling platforms and built prognostic models using machine learning methods. The Kaplan–Meier method and log-rank tests were applied to estimate correlations of the miRNA signature with survival, and the area under the time-dependent ROC curve (AUC) and concordance index (C-index) were used to assess the predictive power of prognostic models. We also investigated the biological roles of the prognostic miRNAs through identifying their regulated genes and association with immune infiltration.

**Results:**

We constructed a prognostic model based on 6-miRNA signature (ebv-miR-BART12, ebv-miR-BART15, miR-29c-3p, miR-30e-5p, hsa-miR-375-3p, has-miR-93-5p) using the elastic net penalized Cox regression model. The AUCs of our model predicting 1-, 3-, and 5-year overall survival rates were 0.90, 0.73, and 0.70 for the external validation dataset and showed better prognostic power than models using previously reported miRNA signatures. The 6-miRNA risk score was an independent prognostic predictor for overall survival (OS), disease-free survival (DFS), and metastasis-free survival (MFS). It could stratify patients into low-risk and high-risk groups; patients in the low-risk group treated with concurrent chemotherapy had a better survival. On the basis that the 6-miRNA risk score improved the current clinical gold standard for prognosis, we built a nomogram integrating both clinical characterizations and the risk score to predict 3-, 5-, and 10-year overall survival. Functional analysis suggested that the six miRNAs mainly play roles in virus infection pathways and oncogenic signaling pathways and associated with B-cell expression.

**Conclusion:**

We identified a 6-miRNA prognostic signature in nasopharyngeal carcinoma across miRNA profiling platforms and patient geographical difference, which showed good prediction capability in terms of OS, DFS, and MFS. The 6-miRNA risk score might be helpful for clinicians to make treatment strategies and predict patient outcomes.

## Introduction

Nasopharyngeal carcinoma (NPC) occurs in the nasopharynx, which is hard to examine and detect early. NPC is rare in Western countries (1–2 cases per 100,000) but has a high incidence rate (10–30 cases per 100,000) in Southeast Asia (Mahdavifar et al., 2016). NPC pathogenesis has been reported to be strongly associated with genetics, EBV infection, and environmental effects (Hildesheim and Wang, 2012). The main treatment for NPC is radiation therapy, and chemotherapy is often combined for patients in the late stage (Chan et al., 2010).

MicroRNAs (miRNAs) are short non-coding RNAs which can regulate gene expression post-transcriptionally and have been implicated as key players in a number of disease processes, including cancer occurrence and progression (Lee and Dutta, 2009; Peng and Croce, 2016). MiRNAs also play roles in the drug resistance of tumor cells by targeting genes related to cell proliferation, cell cycle, and apoptosis (Si et al., 2019). More and more studies proved miRNAs as potential biomarkers for the diagnosis, prognosis, and therapy of human cancers including NPC. Several studies have reported the clinical significance of both human- and EBV-encoded miRNAs in NPC. miR203 and miR-23a proved to be associated with NPC radioresistance (Petersson, 2015; Qu et al., 2015). The expression of miR-9 was reported negatively associated with NPC progression (Lu et al., 2014). Four viral miRNAs (BART5-5p, BART7-3p, BART9-3p, and BART14-3p) could work cooperatively to negatively regulate the expression of the ATM gene in response to DNA damage, which promote the maintenance of viral latency and tumorigenesis of NPC (Lung et al., 2018). Additionally, high-throughput miRNA expression profiles were performed to find miRNA signature related to the prognosis of NPC. Bruce et al. identified a 4-miRNA signature (miR-154, miR-449b, miR-140, and miR-34c) associated with risk of distant metastasis (Bruce et al., 2015). Liu et al. reported a 5-miRNA signature (miR-93, miR-26a, miR-142, miR-29c, and miR-30e) that could predict overall survival and disease/metastasis-free survival, which was independent to TNM stage (Liu et al., 2012). Although those miRNAs have a prognostic power in NPC, there is a scarcity of independent validation for these miRNAs.

In this work, we integrated miRNA expression data from different profiling platforms, identified a robust 6-miRNA prognostic signature through machine learning method, and evaluated it using an independent dataset. The 6-miRNA signature showed better prognostic power than previous miRNA signatures and improved the current clinical gold standard for prognosis. Further analysis showed that the 6-miRNA risk score could help guide precision treatment for NPC patients with concurrent chemotherapy or not. We also explored the biological function roles of the six miRNAs and their potential association with immune infiltration.

## Materials and Methods

### miRNA Expression Profiles and Clinical Data of NPC

We used the keywords “nasopharynx cancer” and “miRNA” to search in EBI ArrayExpress and Gene Expression Omnibus (GEO), then manually reviewed and selected cohorts with both miRNA expression and clinical survival information. Finally, three datasets with at least 50 NPC patients were kept for analysis, which included GSE32960 (Liu et al., 2012), GSE70970 (Bruce et al., 2015), and GSE36682. The three datasets contained 612 patients in total and used three different platforms to measure miRNA expression (Table 1). The miRNA profiling platforms for GSE32960, GSE70970, and GSE36682 were GPL14722 (with 917 miRNA probes), GPL20699 (with 734 miRNA probes), and GPL15311 (with 1,004 miRNA probes), respectively.

**TABLE 1:** Demographics of the three NPC cohorts.

	**Training dataset**		**Test dataset**
	**GSE32960 (*N* = 312)**	**GSE70970 (*N* = 246)**	**GSE36682 (*N* = 62)**
miRNA platform	GPL14722	GPL20699	GPL15311
Country	China	Canada	China
Sex = male (%)	233 (74.7)	175 (71.1)	51 (82.3)
Age, mean (SD)	46.83 (11.0)	50.33 (13.8)	/
WHO type (%)		/	/
Non-keratinizing undifferentiated carcinoma	301 (96.5)	/	/
Non-keratinizing differentiated carcinoma	8 (2.6)	/	/
Keratinizing squamous cell carcinoma	3 (1.0)	/	/
VCA-IgA, mean (SD)	364.07 (335.9)	/	/
EA-IgA, mean (SD)	46.51 (66.3)	/	/
AJCC7-TNM stage (%)			
I	12 (3.8)	23 (9.4)	/
II	86 (27.6)	56 (23.0)	/
III	91 (29.2)	79 (32.4)	/
IV	123 (39.4)	86 (35.2)	/
Concurrent chemotherapy (%)	134 (42.9)	126 (51.2)	/
5-Year overall survival rate (%)*	78.2% (73.6–83%)	77.3% (72–83.1%)	59.4% (58.8–81.8%)
5-Year disease-free survival rate (%)*	69.5% (64.6–74.9%)	68.7% (63.0–75.0%)	/
5-Year metastasis-free survival rate (%)*	78.5% (74.0–83.3%)	86.7% (82.3–91.3%)	/

*Survival rate (confidence interval).

**FIGURE 1:**
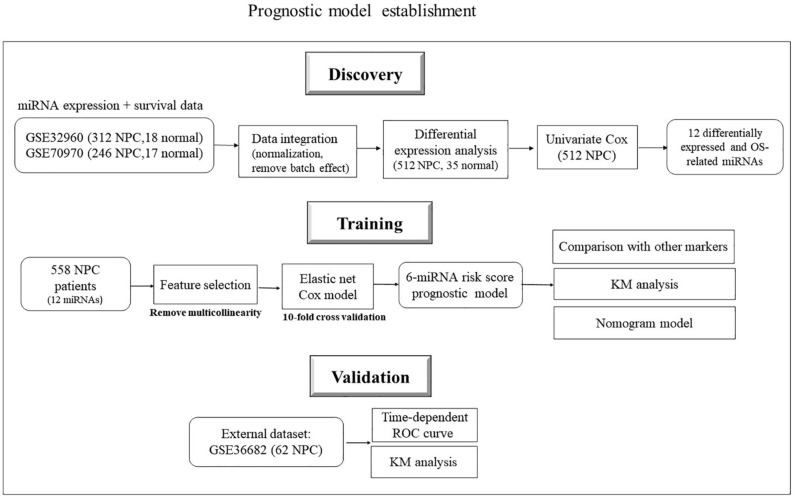
Flowchart of data collection and analysis.

The two big miRNA expression datasets (GSE32960 and GSE70970) were used to build the prognostic model. GSE32960 included 312 NPC patients and 18 normal controls from Sun Yat-sen University Cancer Center, China; GSE70970 included 246 NPC patients and 17 normal controls from Princess Margaret Cancer Centre, Canada. GSE36682, which contained 62 NPC patients from Sun-Yat sen University, China, was used as an independent validation dataset to evaluate model performance (see Figure 1). R 4.0.2 was used for statistical analysis.

### Identification of Differentially Expressed miRNAs

The expression values of many miRNAs were 0 in GSE70970, which indicated severe degradation. Therefore, we only kept miRNAs that were expressed in at least 10% of the samples, and then 337 out of 734 miRNA probes from GSE70970 were left. We merged GSE32960 and GSE70970 through unique miRNA ID and removed batch effects using the “ComBat” method (Leek et al., 2012). Finally, 309 common miRNAs were kept for further differential expression analysis and survival analysis. miRbase (Kozomara et al., 2019) was used to normalize miRNA names from each platform. We used the R package “limma” (Ritchie et al., 2015) to identify differentially expressed (DE) miRNAs with the following criteria: (1) adjusted *p* < 0.05 and (2) absolute fold change > 1.5 between cancer and normal tissues.

### miRNA Feature Identification and Prognostic Model Building

The prognostic power of the expression level of each DE miRNA was evaluated using the univariate Cox regression model and log-rank test. To remove random effects, we repeated the Cox test 1,000 times using bootstrap samples. DE miRNAs, which were statistically significantly associated with overall survival at least 500 times with *p*-value < 0.05, were selected as features for machine learning to build the prognostic model.

We tried three penalized Cox proportional hazard (PH) regression models (ridge regression, elastic net, and lasso) to predict the overall survival rate. 10-fold cross-validation was used to determine the best penalty parameters, and a penalized Cox model was fitted using these optimal parameter values. All the machine learning models were fitted using the R package “glmnet” (Friedman et al., 2010). Time-dependent receiver operating characteristic (ROC) curves (Kamarudin et al., 2017), the area under the ROC curve (AUC), and the concordance index (C-index) were applied to assess the predictive power of prognostic models, which can be estimated using the R packages “timeROC” (Kamarudin et al., 2017) and “pec” (Mogensen et al., 2012).

### miRNA-Targeted Gene Prediction

The putative targets of human miRNAs were obtained from miRTarBase (Chou et al., 2018), in which the miRNA-target interactions (MTIs) are validated experimentally by reporter assay, Western blot, microarray, and next-generation sequencing experiments. The putative targets of EBV miRNAs were predicted using the ‘‘MirTarget’’ prediction algorithm in miRDB^1^ (Wang, 2016).

To narrow down the list of putative target genes of miRNAs, expression correlation analysis between miRNA and the targeted mRNA was applied to select more reliable MTIs using the dataset GSE118721 (Lin et al., 2018), which provided both miRNA and mRNA expression profiles for seven NPC biopsy specimens and four normal nasopharyngeal mucosal specimens. If the target gene of a miRNA was differentially expressed with opposite direction of change and the negative correlation was significant (*p* < 0.05 and *r* < −0.5), the MTI would be regarded as reliable and the target genes would be used for further function analysis.

### Gene Function Enrichment Analysis

We used the R package “clusterProfiler” (Yu et al., 2012) to perform gene ontology and pathway enrichment analysis. Adjusted *p*-values less than 0.01 obtained by the BH method were regarded as statistically significant.

## Results

### Clinical Characterizations of NPC Patients

The three miRNA expression datasets involving 620 NPC patients were from three platforms and two populations (Table 1). For patients from GSE32960 and GSE70970, the clinical characterizations were similar: the average age was about 50 and the predominant (>70%) patients were male, all patients received radiotherapy and nearly half had been treated with concurrent chemotherapy, about 69% were already late stage (TNM stage III or IV), and the 5-year overall survival rate was ∼78%. We integrated these two expression datasets into one as training dataset for model building and another dataset GSE36682 was used as an independent validation dataset for model evaluation. There were 62 patients in the dataset GSE36682, with little clinical information other than overall survival. The 5-year overall survival rate of those 62 patients was 59.4% (CI: 58.8–81.8%).

### 6-miRNA Signature Identification and Prognostic Model Building

After removing degraded miRNAs and batch effects, we merged the two datasets (GSE32960 and GSE70970) into one dataset, which contained the expression profile of 309 miRNAs from 558 NPC patients and 35 normal controls; 112 DE miRNAs were identified. Twelve survival-related DE miRNAs were then selected using the univariate Cox analysis with 1,000 bootstrap samples (see section “Materials and Methods”).

**FIGURE 2:**
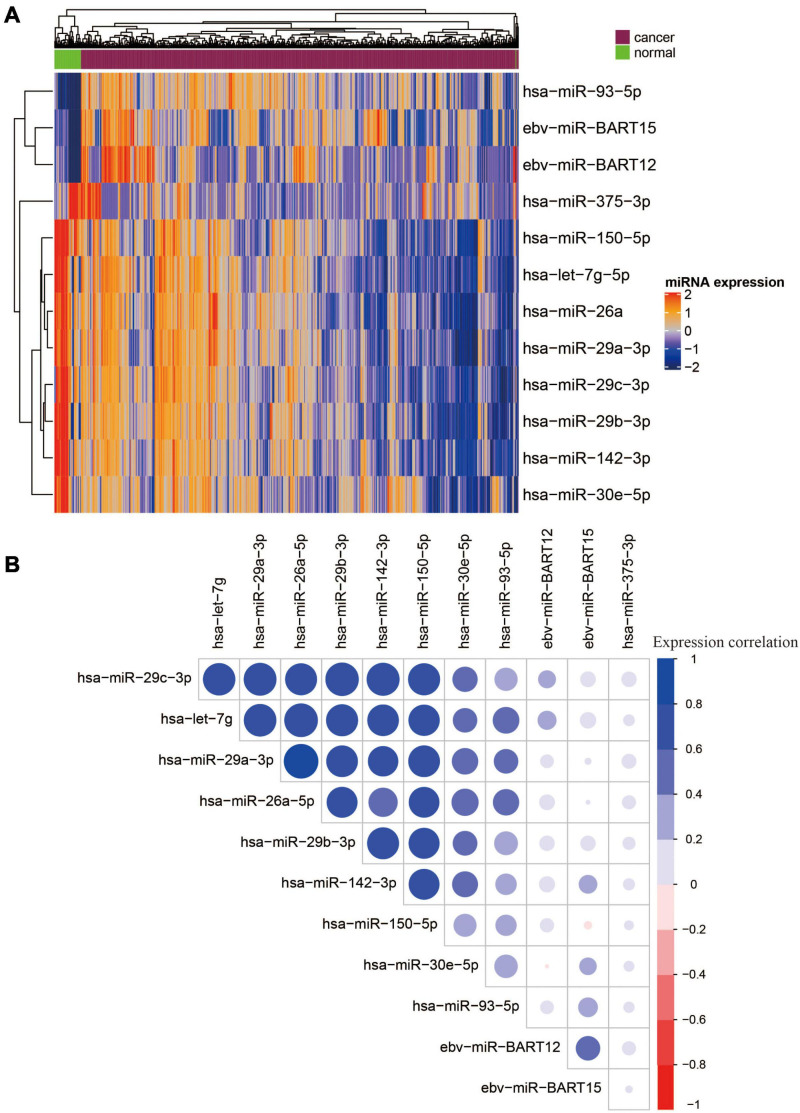
Heatmap and correlation matrix of 12 OS-related DE miRNAs. **(A)** Heatmap of 12 overall survival and differentially expressed miRNAs. **(B)** Correlation matrix of 12 OS-related DE miRNAs.

Among the 12 miRNAs, 3 miRNAs (ebv-miR-BART12, ebv-miR-BART15, and hsa-miR-93-5p) were overexpressed, while the other 9 miRNAs were downregulated (Figure 2A). There were strong correlations between seven miRNAs, namely, hsa-miR-29c-3p, hsa-let-7g, hsa-miR-29a-3p, hsa-miR-26a-5p, hsa-miR-29b-3p, hsa-miR-142-3p, and hsa-miR-150-5p (Figure 2B). To reduce multicollinearity and use fewer features for model building, we only kept hsa-miR-29c-3p as representative since it showed the highest average expression similarity among the seven correlated miRNAs. Finally, six miRNAs, namely, two EBV miRNAs (ebv-miR-BART12 and ebv-miR-BART15) and four human miRNAs (miR-29c-3p, miR-30e-5p, hsa-miR-375-3p, and has-miR-93-5p), were selected for further model building.

To predict overall survival (OS) using the combination of six miRNAs, we assessed three penalized Cox regression models, namely, ridge regression (Hoerl and Kennard, 1970), elastic net (Zou and Hastie, 2005), and lasso (Tibshirani, 1996). Among them, elastic net regression was consistently the best performer, with minimal mean cross-validated error (data not shown). Using the elastic net penalized Cox PH model to miRNA expression data from the training cohort, we obtained an optimal risk assessment model (1) utilizing the regression coefficients of six miRNAs:

Risk⁢score=ebv-miR-BART12*(-0.226)+ebv

-miR-BART15*(-0.220)+hsa-miR-29⁢c-3⁢p

*(-0.377)+hsa-miR-30⁢e-5⁢p*(-0.423)+hsa

-miR-375-3⁢p*(0.502)+hsa-miR-93-5⁢p

(1) *(0.698)

**FIGURE 3:**
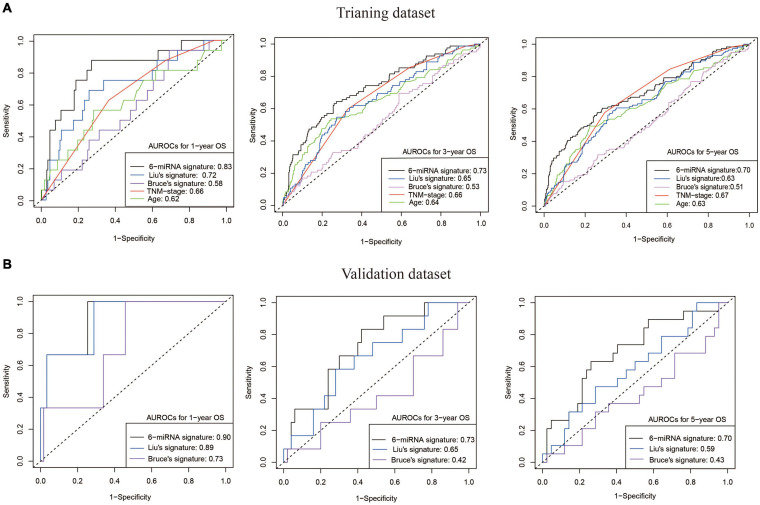
Good OS prediction capability of our 6-miRNA signature model. **(A)** The AUCs of models built by our 6-miRNA signature, the 5-miRNA signature of Liu, the 4-miRNA signature of Bruce, and clinical variables (TNM stage and age) using the training dataset (558 patients). **(B)** The AUCs of models built by our 6-miRNA signature, the 5-miRNA signature of Liu, and the 4-miRNA signature of Bruce using the external validation dataset GSE36682 (62 patients). OS, overall survival.

The risk score (RS) was calculated for each patient in the training (GSE32960 and GSE70970) and independent test cohorts (GSE36682). The overall prognostic accuracy of the RS, assessed as a continuous variable, was investigated using time-dependent ROC analysis at three time points (1, 3, and 5 years). The AUCs of the predicted 1-, 3-, and 5-year overall survival rates were 0.83, 0.73, and 0.70 (training cohort) and 0.90, 0.73, and 0.70 (test cohort), respectively. Then we compared our 6-miRNA signature with the previously reported 5-miRNA signature (miR-93, miR-26a, miR-142, miR-29c, and miR-30e) of Liu et al. (2012) and the 4-miRNA signature (miR-154, miR-449b, miR-140, and miR-34c) of Bruce et al. (2015). Our 6-miRNA signature showed better permanence than previous signatures in both training and test cohorts (Figures 3A,B). The 6-miRNA signature also exhibited better prognostic power than TNM stage and age (Figure 3A).

**FIGURE 4:**
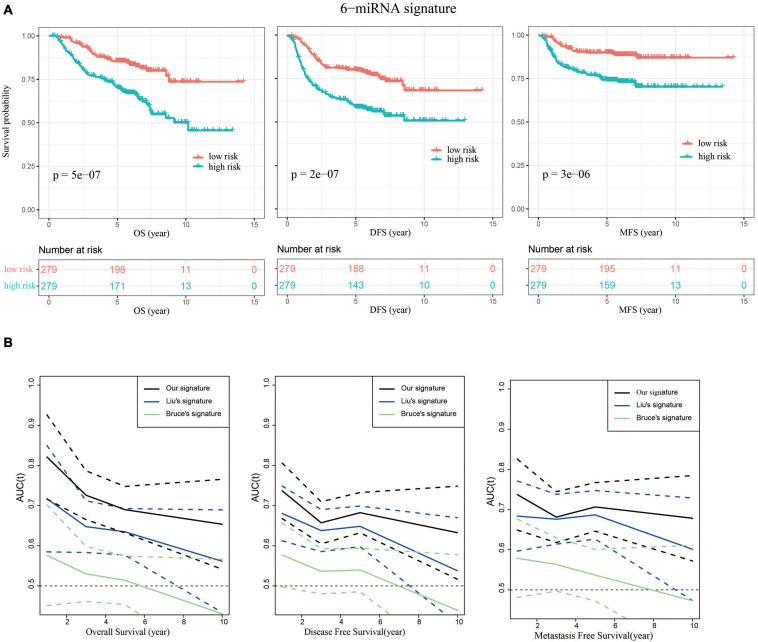
Our 6-miRNA signature could predict OS, DFS, and MFS. **(A)** Kaplan–Meier graphs depicting overall survival (OS), disease-free survival (DFS), and metastasis-free survival (MFS) using the training dataset stratified by the 6-miRNA risk score, and *p*-values were based on log-rank test. **(B)** Comparison of the AUCs using our signature and the signatures of Liu and Bruce to predict OS, DFS, and MFS. The dashed lines represent the corresponding 95% confidence intervals of AUC by each model.

We then evaluated the prognostic power of 6-miRNA risk scores to predict disease-free survival (DFS) and metastasis-free survival (MFS). NPC patients were divided into high-risk and low-risk groups based on the median value of risk scores (0.44). The low-risk group had significantly better OS, DFS, and MFS, indicating the 6-miRNA signature could be used as a good prognostic biomarker (Figure 4A). Moreover, compared with the signatures of Liu and Bruce, the AUCs of prognostic models using our 6-miRNA signature were also higher in terms of MFS and DFS (Figure 4B).

**FIGURE 5:**
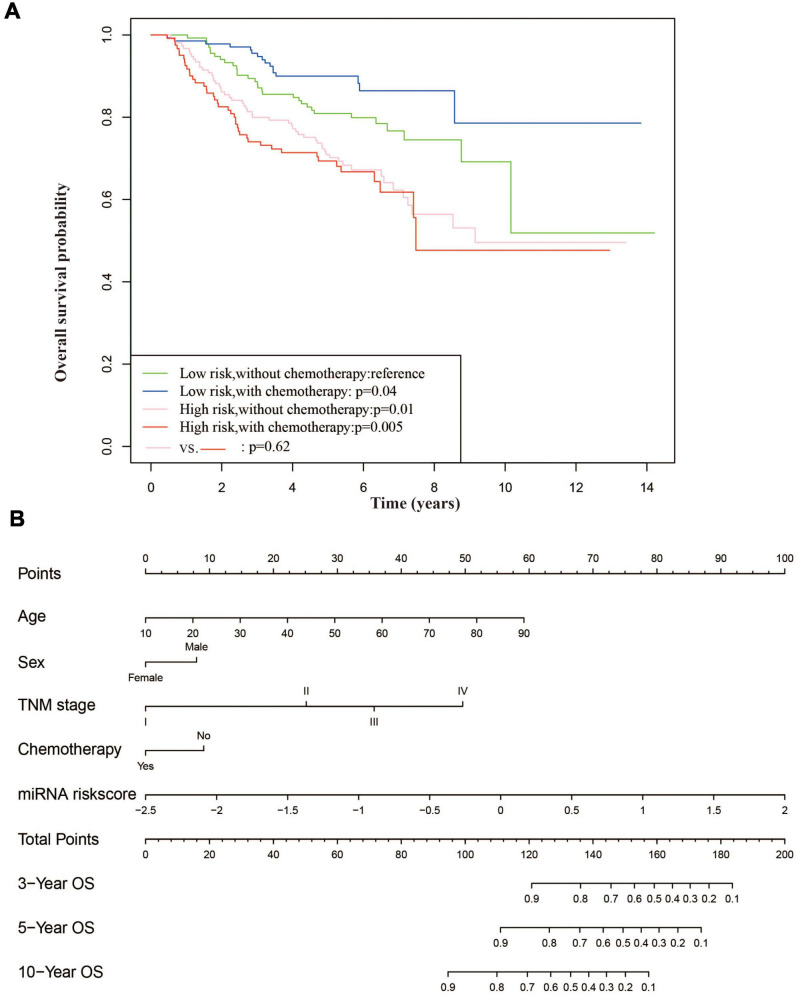
The 6-miRNA risk score could be used to guide chemotherapy and a nomogram for OS prediction based on clinical characterization and 6-miRNA risk score. **(A)** Survival plots of patient stratified by 6-miRNA risk scores and chemotherapy treatment. Patients in the low-risk group treated with chemotherapy had better overall survival. **(B)** Construction of a nomogram based on clinical characterization and 6-miRNA risk score to predict the 3-, 5-, and 10-year overall survival of NPC patients.

Radiotherapy alone is not sufficiently effective for patients with advanced NPC, and more and more studies suggest that the addition of concurrent chemotherapy to radiotherapy significantly improves survival in those NPC patients (Blanchard et al., 2015). In our study, patients in the low-risk group treated with concurrent chemotherapy showed significantly better overall survival than those without concurrent chemotherapy (*p* = 0.038), while for the high-risk group, there are no significant differences in the survival between patients with and without chemotherapy (*p* = 0.62) (Figure 5A). Low-risk patients also benefited from concurrent chemotherapy in terms of DFS and MFS (data no shown), which suggested that 6-miRNA risk score could be helpful for precision treatment. Considering that age was an important risk factor for NPC, we did an exploratory subset analysis by age. It was noticed that the 6-miRNA prognostic model exhibited higher AUCs in the younger group than in the older group (see Supplementary Figure 1).

### Construction of a Nomogram Based on the 6-miRNA Risk Score and Clinical Variables

To evaluate whether the 6-miRNA risk score could be an independent prognostic predictor, multivariate Cox regression analyses were conducted. After adjusted by clinical characteristics including age, gender, concurrent chemotherapy, and TNM stage, the risk score of 6-miRNA was still significantly associated with OS, DFS, and MFS (see Supplementary Figure 2). Moreover, the 6-miRNA signature significantly improved the prediction accuracy of the multivariate Cox models for OS, DFS, and MFS when combined with clinical variables (Table 2).

**TABLE 2:** Comparison of the survival models without and with 6-miRNA risk score using 558 patients.

	**Without 6-miRNA (only clinical variables)**	**With 6-miRNA (combined with clinical variables)**
Overall survival	0.68 (0.63–0.72)	0.76 (0.71–0.78)
Disease-free survival	0.64 (0.60–0.68)	0.72 (0.67–0.74)
Metastasis-free survival	0.63 (0.56–0.69)	0.73 (0.65–0.77)

*The concordance index (C-index) and its confidence interval were estimated from the Cox models. Higher C-index indicates better predictive performance.*

Based on multivariate Cox analysis, a nomogram which integrated the 6-miRNA risk score and other clinical variables was generated to predict the probability of 3-, 5-, and 10-year overall survival for NPC patients using 558 patients (Figure 5B). The nomogram showed adequate discrimination ability in internal validation with a C-index of 0.76 (95% CI: 0.71–0.78).

### Functional Roles of Six miRNAs

**FIGURE 6:**
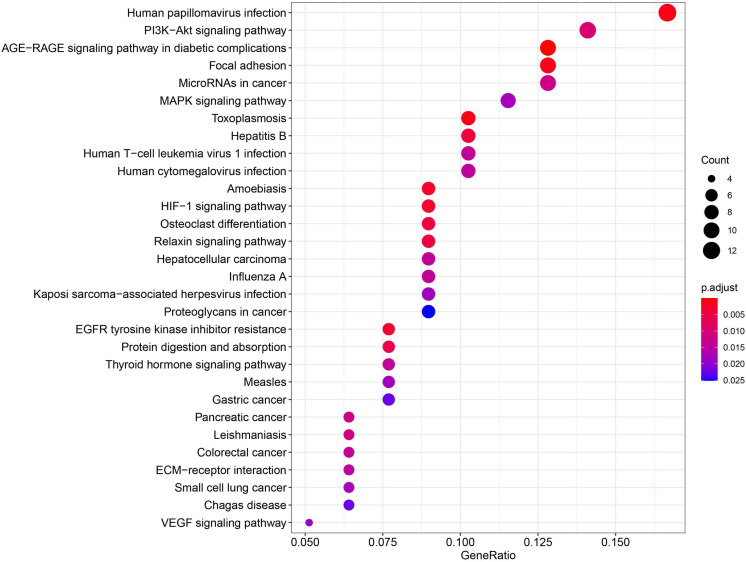
The predicted 6-miRNA target genes were mainly enriched in virus infection pathways and cancer-related pathways.

Using miRNA-target interactions from miRTarBase, we obtained 3,073 MTIs for miR-93-5p, miR-30e-5p, miR-29c-3p, and miR-375-3p. By using the “MirTarget prediction” algorithm in miRDB, we predicted 603 EBV miRNA-targeted host gene pairs. To further confirm the list of putative target genes of the six DE miRNAs in NPC, we checked whether a negative correlation existed between the miRNA and its targeted mRNA using the dataset GSE118721 (see section “Materials and Methods”). Through co-expression analysis, we finally got 152 likely MTIs (Supplementary Table 1), in which the miRNA negatively regulated its corresponding genes. Six overexpressed oncogenes including *BCL2*, *NOTCH1*, *SOX4*, and *CTNNB1* and 10 downregulated tumor suppressor genes including *PRKCB*, *IRF1*, *CYLD*, and *TGFB1* were identified as the targets of the DE miRNAs (Table 3), which may promote cancer development. Functional analysis of the predicted six miRNA-targeted genes showed enrichment in virus infection-related pathways like human papillomavirus infection and cancer-related pathways like PI3K–Akt signaling pathway, focal adhesion, and MAPK signaling pathway (Figure 6).

**TABLE 3:** miRNA-targeted tumor suppressor genes and oncogenes.

**miRNA**	**Target**	**Correlation**	**DE miRNA**	**DE target gene**	**Target gene class**
hsa-miR-29c-3p	***BCL2***	0.66	Down	Up	Transporter
hsa-miR-29c-3p	***WWTR1***	0.53	Down	Up	Transcription regulator
hsa-miR-30e-5p	***NOTCH1***	0.63	Down	Up	Transcription regulator
hsa-miR-30e-5p	***SOX4***	0.54	Down	Up	Transcription regulator
hsa-miR-375-3p	***CTNNB1***	0.69	Down	Up	Transcription regulator
hsa-miR-375-3p	***MST1R***	0.59	Down	Up	Kinase
hsa-miR-93-5p	*PRKCB*	0.85	Up	Down	Kinase
hsa-miR-93-5p	*EZH1*	0.85	Up	Down	Enzyme
hsa-miR-93-5p	*KLF6*	0.83	Up	Down	Transcription regulator
hsa-miR-93-5p	*IRF1*	0.78	Up	Down	Transcription regulator
hsa-miR-93-5p	*TGFB1*	0.76	Up	Down	Growth factor
hsa-miR-93-5p	*TUSC2*	0.75	Up	Down	Other
hsa-miR-93-5p	*CYLD*	0.74	Up	Down	Transcription regulator
hsa-miR-93-5p	*TCF4*	0.72	Up	Down	Transcription regulator
hsa-miR-93-5p	*BTG2*	0.53	Up	Down	Transcription regulator
hsa-miR-93-5p	*PHC3*	0.52	Up	Down	Transcription regulator

*Genes marked in bold are oncogenes, while genes underlined are tumor suppressor genes. DE, differentially expressed; Down, downregulated in cancer compared with normal; UP, overexpressed in cancer compared with normal.*

Solid tumors are commonly infiltrated by immune cells. The tumor-infiltrating immune cells play important roles in maintaining chronic inflammation and promote tumor growth (Pages et al., 2010). In our work, the association of six miRNAs with the immune infiltration was also explored. We performed cell-type enrichment analysis from gene expression dataset GSE118721 using xCell (Aran et al., 2017) to estimate the presence of different immune cells in tumor and calculate an immune score, then did correlation analysis to investigate the relationships between the six miRNAs and immune cells. It was found that miR-29c-3p, miR-30e-5p, and miR-93-5p were significantly associated with immune score (Pearson correlation coefficients: 0.87, 0.69, and 0.71, respectively; *p* < 0.05). Further cell-type analysis showed that tumor suppressors miR-29c-3p and miR-30e-5p were positively correlated with B-cell score, while oncogenic miR-93-5p was negatively associated with B-cell score. It was suggested that the three miRNAs may play roles in regulating the immune microenvironment.

## Discussion

To construct a robust prognostic model regardless of miRNA platforms and the clinical and geographical differences of patients, we integrated the two biggest datasets (GSE32960 and GSE70970) from different population cohorts to identify robust prognostic miRNAs. Through the elastic net penalized Cox regression, we constructed a prognostic model based on six miRNAs to predict OS, which showed good predictive capability in both training and external test datasets and better prognostic power than the previous 5-miRNA signature of Liu and 4-miRNA signature of Bruce (Figure 3). Multivariate Cox analysis showed that 6-miRNA risk score was an independent predictor for OS, DFS, and MFS. Prognostic models combining clinical variables with 6-miRNA risk score exhibited better prediction accuracy than those models without 6-miRNA risk score (Table 2). It was noticed that our 6-miRNA model performed worse using older NPC patients than younger patients, which may be associated with increasing transcriptional noise of old samples (Enge et al., 2017). Furthermore, NPC patients could be stratified into low-risk and high-risk groups based on 6-miRNA risk score, and only patients in the low-risk group could benefit from concurrent chemotherapy (Figure 5A), which could guide the precise treatment of NPC.

As we know, both EBV and human miRNAs play important roles in NPC carcinogenesis and could be drug targets or prognostic biomarkers. The literature review showed that all six miRNAs have been reported to be associated with carcinogenesis in NPC or other cancers (Choi et al., 2013; Liu et al., 2013; Li et al., 2015; Choi and Lee, 2017; Ma et al., 2018; Wu et al., 2020; Jia-Yuan et al., 2020). EBV-encoded miRNA BART12 could promote cell migration and invasion of EBV-associated NPC and gastric cancer by inhibiting *TPPP1* mRNA and activating the cellular EMT process (Wu et al., 2020), and miR-BART15-5p could target BRUCE mRNA and *TAX1BP1* gene in cancer cells and increase apoptosis and chemosensitivity to 5-FU (Choi et al., 2013; Choi and Lee, 2017). Overexpression of miR-93-5p was associated with tumor progression, metastasis, and poor prognosis in head and neck squamous cell carcinoma (HNSCC) (Li et al., 2015). miR-30e-5p inhibits the proliferation and metastasis of nasopharyngeal carcinoma cells by targeting USP22 (Ma et al., 2018). Downregulation of miR-29c-3p promoted NPC cell migration and invasion by targeting TIAM1 (Liu et al., 2013); miR-375-3p plays roles as a tumor suppressor by targeting oncogene PDK1, which promotes the proliferation, migration, and invasion of NPC cells (Jia-Yuan et al., 2020).

Previous signatures only contained human miRNAs, but our 6-miRNA signature included two EBV miRNAs and four human miRNAs. Three miRNAs (ebv-miR-BART12, ebv-miR-BART15, and hsa-miR-375-3p) were not included in previously reported prognostic miRNA signatures, but provided additional information to predict NPC prognosis. MiR-BART15-3p, miR-30e-5p, and miR-29c-3p have been reported to be associated with chemotherapy sensitivity in other cancers (Choi et al., 2013; Choi and Lee, 2017; Liu et al., 2017; Huang et al., 2018), which may explain the association between the 6-miRNA risk score and the response to chemotherapy. Functional analysis of the 6-miRNA target genes showed that they mainly play roles in virus infection pathways and oncogenic signaling pathways (Figure 6), and many target genes were transcriptional factors associated with carcinogenesis, which may magnify the regulation effect of the six miRNAs (Table 3). It was also found that miR-29c-3p and miR-30e-5p were positively associated with B-cell expression, while miR-93-5p was negatively correlated, indicating they may be involved in regulating B-cell-related pathways.

The high expression of oncogene/tumor suppressor is generally thought to be associated with higher/lower death risk in tumors. However, some oncogenes have a paradoxical function in cancer: though they have strong transforming and tumor-promoting properties, they are prognostic markers for favorable survival. This kind of oncogenes is defined as “good oncogenes” (Lee, 2011). In our risk model, it was also found that ebv-miR-BART12 and ebv-miR-BART15 and oncogenes miR-29c-3p and miR-30e-5p were negatively correlated with risk score, seeming to be good oncogenes. The four miRNAs may promote primary tumor development and inhibit mortality through activating the immune system.

Compared with previous miRNA signatures, our work had several advantages: (1) a larger sample size for the training model, covering different populations and platforms: 558 samples for our 6-miRNA signature vs. 156 for the miRNA signature of Liu and 125 for the miRNA signature of Bruce; (2) using an independent external dataset to evaluate the model while the work of the other two signatures did not perform an independent validation using an external dataset; and (3) considering both human miRNAs and EBV miRNAs as prognostic biomarkers. However, there were also some limitations in our study: (1) the independent validation dataset GSE36682 (*n* = 62) was small and did not have complete clinical and survival information, which means that a larger cohort is needed to examine the robustness of the developed signature and nomogram; and (2) despite function analysis indicating that the miRNAs were associated with some critical signaling pathways and immune microenvironment regulation, further experiments are needed to confirm the results of function analysis.

In conclusion, our work constructed a robust prognostic model across miRNA profiling platforms and patient populations and then built a nomogram integrating both 6-miRNA risk score and clinical features, which can be used to predict the OS of NPC patients. Our work would help clinicians develop individualized therapy based on 6-miRNA risk score.

## Data Availability Statement

The original contributions presented in the study are included in the article/Supplementary Material, further inquiries can be directed to the corresponding authors.

## Author Contributions

YL, HL, and YC conceived the study. YC analyzed the data. ZW provided suggestions about the study design and discussion. All authors participated in writing the manuscript, read, and approved the final manuscript.

## Conflict of Interest

The authors declare that the research was conducted in the absence of any commercial or financial relationships that could be construed as a potential conflict of interest.

## Footnotes

1 http://mirdb.org/custom.html

## Supplementary Material

Supplementary Figure 1 The performance of our 6-miRNA prognostic model to predict OS in different age group. **(A)** The AUCs of our 6-miRNA prognostic model to predict OS in age ≤54 and age >54. **(B)** Compare the AUCs of our 6-miRNA prognostic model to predict OS in different age group. The dash lines represent pointwise confidence intervals.

Supplementary Figure 2 The 6-miRNA risk score was an independent predictor for OS, DFS, and MFS. Multivariate Cox analysis evaluating independently predictive ability of 6-miRNA risk score and other clinical risk factors for OS, DFS, and MFS using 558 NPC patients. The square data markers indicate estimated hazard ratios. The error bars represent 95% CIs. OS: overall survival; DFS: disease-free survival; MFS: metastasis-free survival.

Supplementary Table 1 Target genes of the prognostic miRNAs.
