# Supplementary figures and images for: Integrative Analysis Identified a 6-miRNA Prognostic Signature in Nasopharyngeal Carcinoma
Source: Front Cell Dev Biol. 2021 Jul 16;9:661105. doi: 10.3389/fcell.2021.661105 (PMC8322954; doi:10.3389/fcell.2021.661105)

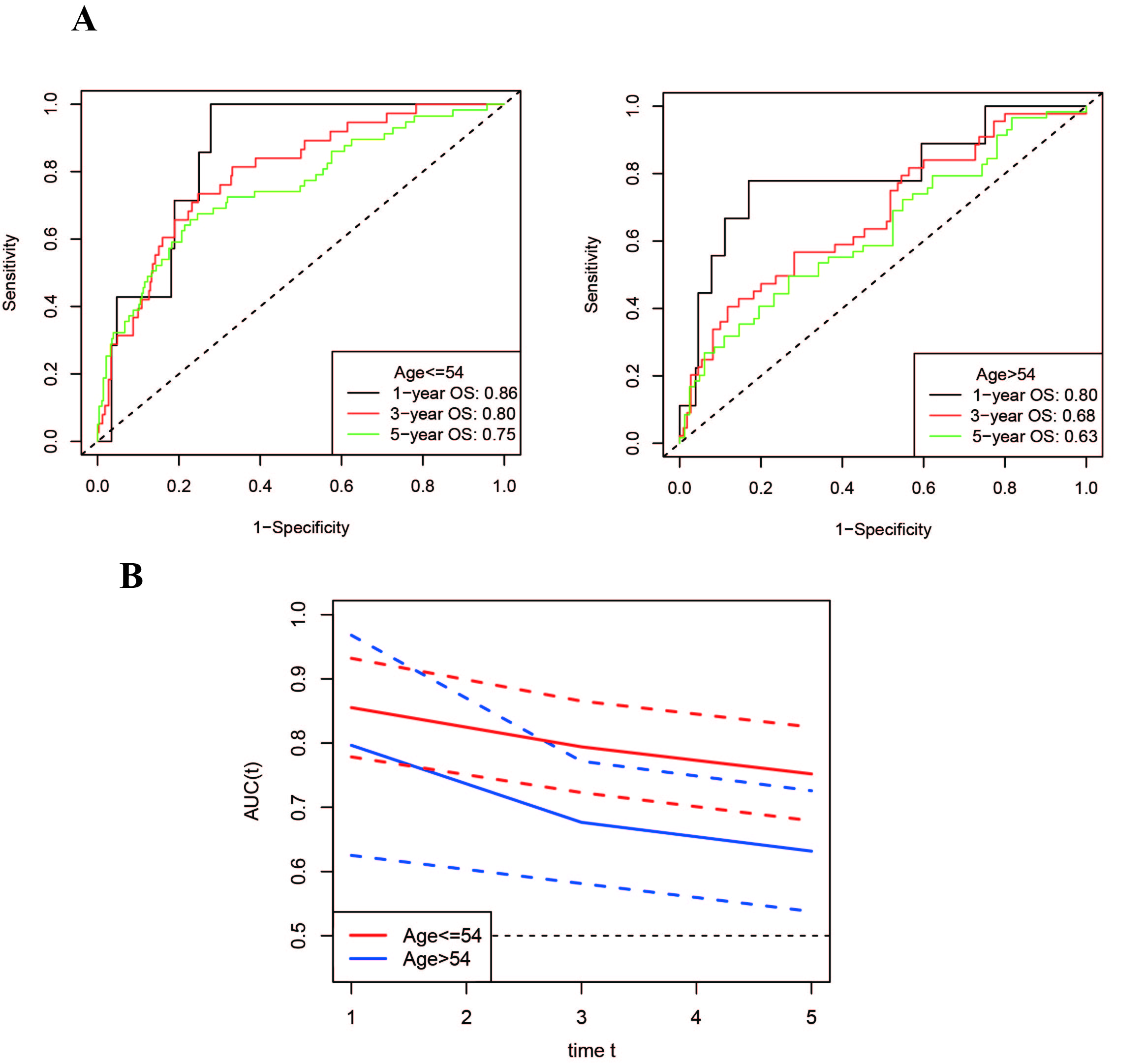

Supplement: Supplementary Figure 1 — The performance of our 6-miRNA prognostic model to predict OS in different age group. (A) The AUCs of our 6-miRNA prognostic model to predict OS in age ≤54 and age >54. (B) Compare the AUCs of our 6-miRNA prognostic model to predict OS in different age group. The dash lines represent pointwise confidence intervals. [file Image_1.JPEG]

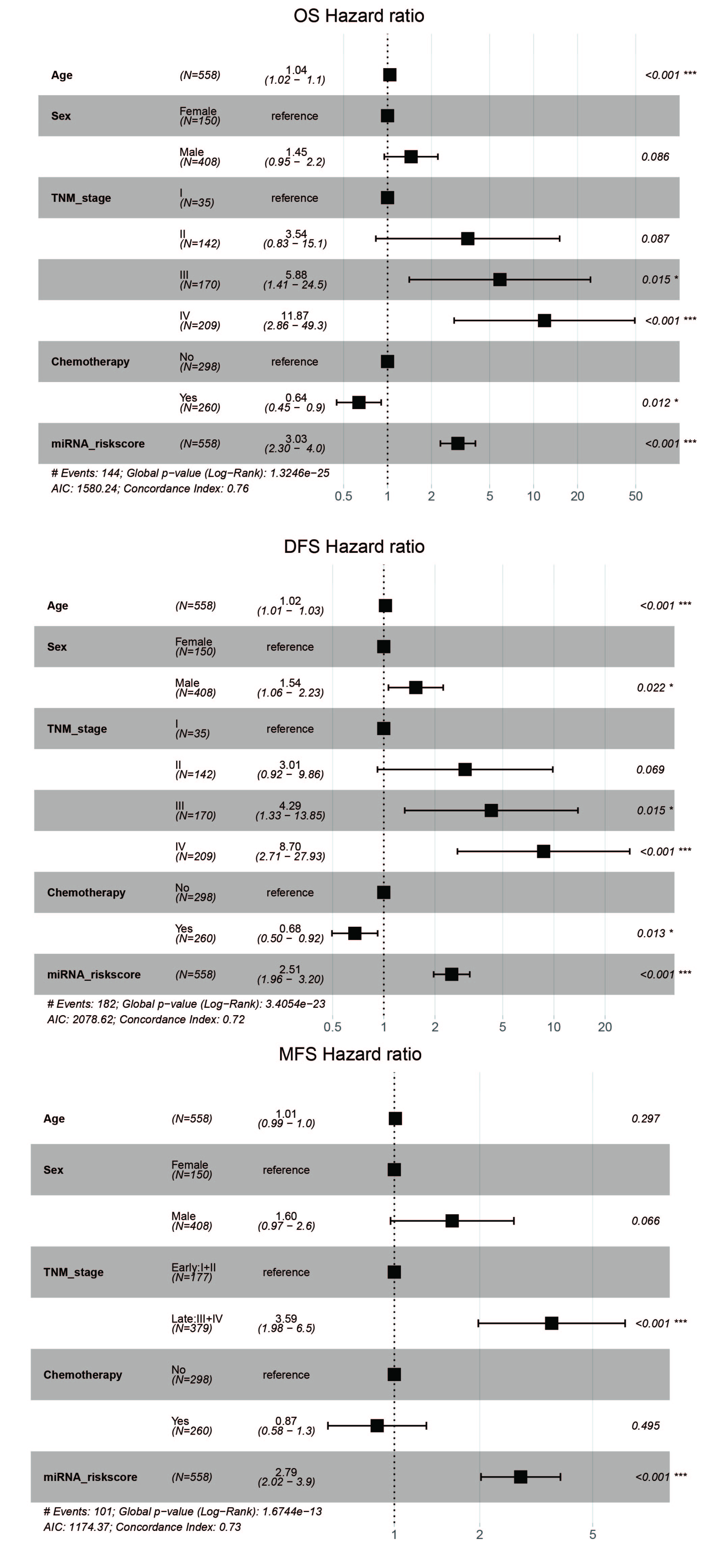

Supplement: Supplementary Figure 2 — The 6-miRNA risk score was an independent predictor for OS, DFS, and MFS. Multivariate Cox analysis evaluating independently predictive ability of 6-miRNA risk score and other clinical risk factors for OS, DFS, and MFS using 558 NPC patients. The square data markers indicate estimated hazard ratios. The error bars represent 95% CIs. OS: overall survival; DFS: disease-free survival; MFS: metastasis-free survival. [file Image_2.JPEG]
